# Augmentation of the Sympathetic Skin Response after Electrical Train Stimuli

**DOI:** 10.3389/fneur.2012.00152

**Published:** 2012-10-30

**Authors:** A. Emmer, S. Mangalo, M. E. Kornhuber

**Affiliations:** ^1^Department of Neurology, Martin-Luther-University Halle-WittenbergHalle, Germany

**Keywords:** sympathetic skin response, augmentation, temporal summation, habituation, train stimuli

## Abstract

It is well known that the size of the sympathetic skin response (SSR) depends on the stimulus strength. In the present investigation train stimuli (TS) were employed to study the behavior of the SSR when recruited above the usual level. The SSR was obtained in healthy human subjects over the palm of the hand after supramaximal single stimuli (SS) and trains of three (TS; interstimulus interval 3 ms) over the ipsilateral superficial radial nerve in 15 healthy volunteers. Ipsilateral to the stimulus site SSR amplitudes were 5.7 ± 5.3 (SS) and 7.7 ± 5.9 mV (TS; *p* < 0.001), and contralateral 6.3 ± 6.3 (SS) and 7.2 ± 4.9 mV (TS; not significant). The relative gain in amplitude after TS vs. SS was negatively correlated with the SSR amplitude after SS ipsilateral (*p* < 0.0005) and contralateral to the stimulus site (*p* < 0.01). The increase in SSR amplitudes after TS compared with SS is in line with temporal summation of the excitatory synaptic input in neurons generating the SSR. Driving the SSR with TS is of possible relevance for the investigation of disorders of the peripheral or central autonomic nervous system.

## Introduction

**Figure 1:**
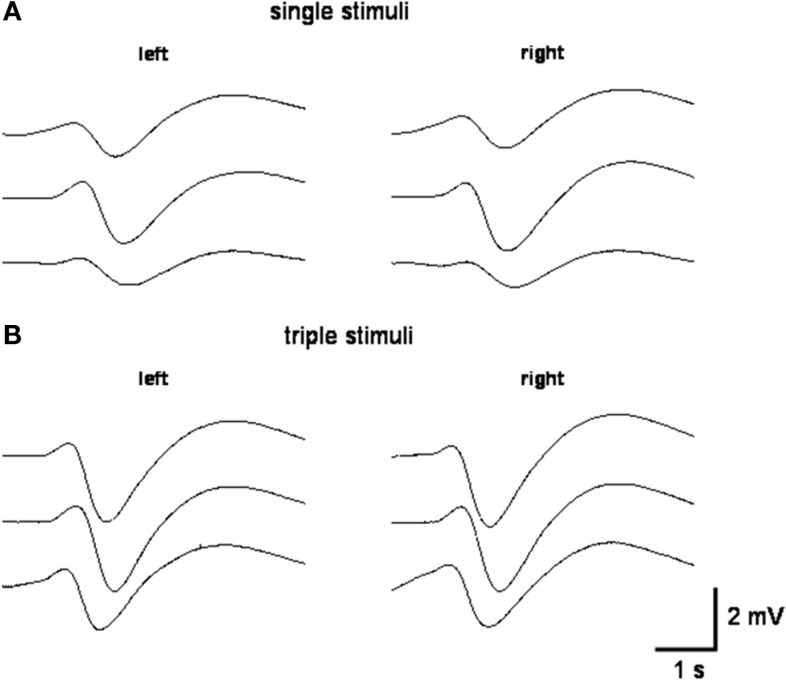
**Representative SSR recordings obtained after single stimuli (A) and triple stimuli (B) over the right superficial radial nerve at the wrist of the same subject**.

The sympathetic skin response (SSR) is one of the most important and intensively studied neurophysiological investigations of the autonomous nervous system. The method is easy to perform (Shahani et al., 1984). Normal values have been repeatedly given in the literature (for review see Figure 1; Litchy, 2008).

In healthy subjects the SSR amplitudes mainly depend on vigilance (Ligouri et al., 2000), stimulus strength (Toyokura, 2006), and on the novelty of the stimulus including the influence of habituation (Hoeldtke et al., 1992). When SSR are elicited by electrical skin stimuli, the stimulus strength is limited by the number of afferent neurites. In case all neurites are stimulated to give rise to action potentials, further increase of the stimulus current usually does not result in an increase of afferent information. However, the latter may be increased further by a sequence of afferent action potentials with a short time interval that may result in temporal summation of excitatory postsynaptic potentials at the dendritic tree of neurons involved in generating the SSR. Therefore, in the present investigation the stimulus strength was elevated in a well defined way by using electrical train stimuli (TS) with a short interstimulus interval to characterize the SSR response compared with single stimuli (SS).

## Materials and Methods

Informed consent was obtained from all volunteers examined. The Ethical committee approval was granted for this investigation from the Ethics committee of the University Hospital of the Martin-Luther-University Halle-Wittenberg, Germany. The SSR was obtained with surface electrodes over the palm vs. the back of the hand after SS and TS over the right superficial radial nerve at the wrist with an interstimulus interval of 3 ms in 15 healthy volunteers at the age between 21 and 45 years. The Multiliner E-System^®^ and Multiliner E Software (Viasys Höchberg, Germany) was used. The recording electrode was placed on the palm, while the reference electrode was placed on the back of the hand. Filter settings were 0.05 Hz and 40 Hz. Three SS were delivered without warning at intervals of 2–4 min. After a break of 10 min, to recover the starting conditions three TS were given at similar time intervals. SSR N1/P1-amplitudes were measured throughout. All experiments were done by the same examiner at a similar daily time window between 5 and 7 p.m. in the evening, in a quiet, semidark room, with a constant temperature of 24.0°C and humidity of 30–35%. Skin temperature was between 31°C and 32°C. Subjects were asked to remain calm and quiet throughout the stimulating procedure.

### Statistical methods

The non-parametric Friedman ANOVA was used to test for statistical significant differences when results obtained after the first, the second, and the third stimulus were compared. Wilcoxon’s test was then taken *post hoc*. Wilcoxon’s test was furthermore used to compare the amplitudes obtained after SS and TS. *p*-Values below 0.05 were considered as significant.

## Results

**Figure 2:**
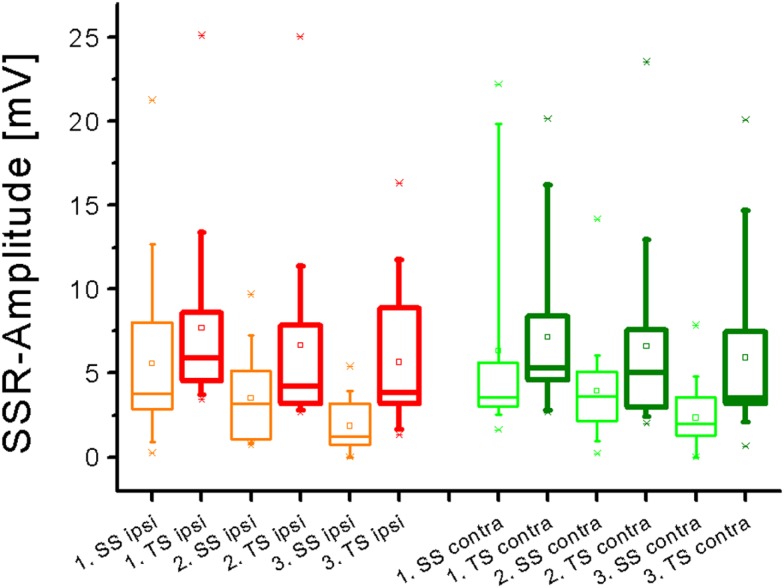
**Box plot diagram summarizing SSR N1-P1-amplitude values obtained in the entire sample after single stimuli (SS) and train stimuli (TS) delivered over the right superficial radial nerve**. A significant amplitude decline was obtained on either side with SS but not with TS (Friedman test).

In almost all instances SSR could be obtained in the 15 subjects after each of three SS and TS from the ipsilateral and contralateral hand, respectively (Figure 2). There was no statistically significant difference of SSR amplitude values between both sides, irrespective of stimulus type (SS or TS). Also when the sum of the SSR amplitude values after all three stimuli were taken together, no amplitude difference between the sides became detectable.

**Figure 3:**
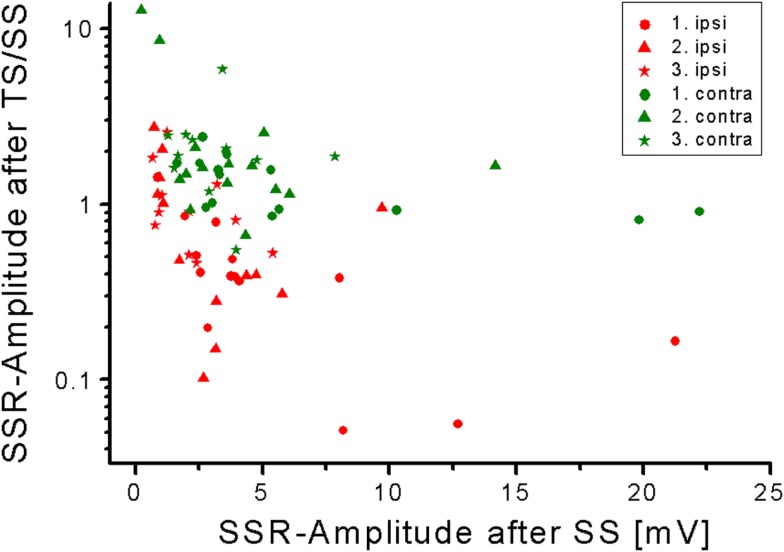
**Negative relation of SSR amplitudes measured after TS/SS compared with SS**. Note the higher SSR-amplitude-gain contralateral obtained after train stimuli in comparison to single stimuli.

Sympathetic skin response amplitude values were significantly larger after TS as compared with SS on either hand side (Figure 2; *p* all <0.005, Wilcoxon’s test), except for the response following the first stimulus contralateral to the stimulus site. When the gain in amplitude was plotted as the ratio of SSR after TS divided by the according responses obtained after SS, it became obvious that this gain was larger contralateral to the stimulus site than ipsilateral (Figure 3). This difference was statistically significant when all responses after the first, second, and third stimulus were included in the analysis (*p* < 0.05; Wilcoxon’s test). Furthermore, there was a negative relation of SSR amplitudes measured after TS compared with SS (Figure 3). This was significant ipsilateral (*p* < 0.000001) and contralateral (*p* < 0.002) to the stimulus site (Spearman correlation analysis).

Sympathetic skin response amplitudes declined significantly on the stimulus site from the preceding to the next SS (*p* < 0.01, Friedman test; *p* < 0.05, Wilcoxon’s test) but not from the preceding to the next TS, respectively. Thus, the decremental response with subsequent stimuli was less large after TS than after SS, as can also be seen from Figure 2. Contralateral to the stimulus site, a significant amplitude reduction was only observed after the third as compared to the second SS (*p* < 0.05, Friedman test; *p* < 0.05, Wilcoxon’s test), and no additional significant change was noted between the responses obtained after the first and second SS or within the SSR amplitude-results after TS.

## Discussion

The SSR is widely used among other tests for routine purposes to assess the integrity of peripheral and central autonomous functions. The normal values measured for amplitude and latency of the SSR obtained in the present study are well within previously given figures (Shahani et al., 1984; Knecevic and Bajada, 1985; Drory and Korczyn, 1993; Rossi et al., 2002; Kucera et al., 2004; Takebayashi et al., 2004; Toyokura, 2004, 2006; Lactin et al., 2005). Interestingly, in the present investigation a significant gain in amplitude was found after trains of three stimuli with a short interstimulus interval of 3 ms as compared with SS. Although it is known that SSR amplitudes depend on stimulus strength (Hoeldtke et al., 1992; Kira et al., 2001; Toyokura, 2006), no attempt has been made previously to increase this stimulus strength further by using TS. When the SS current is increased, a growing number of afferent sensory fibers generate action potentials resulting in augmentation of the SSR by spatial summation of afferent action potentials (Hoeldtke et al., 1992; Kira et al., 2001; Toyokura, 2006). This amplitude increase is limited by the number of recruited afferent neurites, however. In the present study, supramaximal sensory stimuli were employed so that all myelinated afferents were already involved in generating the SSR. By using TS with a short interstimulus interval, SSR amplitudes were shown to be significantly increased compared with SS. Obviously TS result in a stronger drive of the involved neurons to generate action potentials, presumably by way of temporal summation of afferent excitatory postsynaptic potentials. An interstimulus interval of 3 ms was taken as this time interval exceeds the refractory period of an axon and at the same time is short enough to allow temporal summation of postsynaptic potentials to occur. It cannot be completely ruled out that recruiting additional non-myelinated nerve fibers by TS also contributed to the observed gain in SSR amplitudes. Furthermore, stimuli were not repeated at a constant rate in order to maintain a certain level of novelty and unpreparedness of the subject. This fact may also have contributed to relatively large SSR amplitudes. Nevertheless, stimulus repetition protocols were the same for SS ant TS, so that differences in amplitude cannot be attributed to differences in stimulus recurrence.

The SSR amplitude-augmentation was significantly larger in the case of SSR of low amplitudes obtained after SS. This relation may be due to the fact, that eventually there are limited numbers of sweat glands available to take part in forming the SSR. When only few sweat glands are recruited by SS, then comparatively large additional numbers of sweat glands may be recruited by way of TS. When, however, a large number of sweat glands is already recruited by SS, the number of sweat glands that can be additionally activated by TS may be small. This inference is supported by the fact that the gain in SSR amplitudes from SS to TS was significantly larger when the amplitudes after SS were lower, i.e., contralateral as compared to ipsilateral to the stimulus site (*p* < 0.05; Figure 3). Alternatively, it cannot be excluded that TS are better capable than SS to activate neurons located contralateral to the stimulus site to generate the SSR.

Sympathetic skin response amplitudes in the present study showed a statistically significant decline when responses to subsequent stimuli were compared. This data confirms previous reports on habituation of the SSR investigated using SS alone (Cariga et al., 2001; Donadio et al., 2005). Furthermore, the excitation exerted by TS resulted in a diminished amplitude decline after subsequent stimuli (Figure 2). Thus, the plus in excitation obviously competes with habituative influences which can be partially overcome by using TS. This difference in the effect of TS as compared with SS needs substantiation in future studies as the delay between the subsequent stimuli was not well standardized and varied between 2 and 4 min. Previously, strong electrical, magnetic, or auditory stimuli have been employed at relatively short interstimulus intervals of 40–60 s to study SSR wave forms (Toyokura, 2012). As the TS used in the present investigation are markedly stronger than the single electrical stimuli used by Toyokura, it would be interesting to combine short stimulus intervals below 1 min with electrical TS.

Taken together, TS can be used to augment SSR significantly. It has to be elucidated further whether this SSR-augmentation phenomenon will prove to be clinically meaningful.

## Conflict of Interest Statement

The authors declare that the research was conducted in the absence of any commercial or financial relationships that could be construed as a potential conflict of interest.

## Abbreviations

MS, millisecond; SS, single stimuli; SSR, sympathetic skin response; TS, train stimuli.
